# The role of N-acetylcysteine in osteogenic microenvironment for bone tissue engineering

**DOI:** 10.3389/fcell.2024.1435125

**Published:** 2024-07-11

**Authors:** Haowen Zheng, Jiacheng Liu, Lanxin Sun, Zhaosong Meng

**Affiliations:** ^1^ School of Dentistry, Tianjin Medical University, Tianjin, China; ^2^ Department of Prosthodontics, Tianjin Medical University School and Hospital of Stomatology, Tianjin, China; ^3^ Department of Oral and Maxillofacial Surgery, Tianjin Medical University School and Hospital of Stomatology, Tianjin, China

**Keywords:** N-acetylcysteine, oxidative stress, tissue engineering, bone regeneration, stem cells, innate immunity, biomaterials

## Abstract

Bone defect is a common clinical symptom which can arise from various causes. Currently, bone tissue engineering has demonstrated positive therapeutic effects for bone defect repair by using seeding cells such as mesenchymal stem cells and precursor cells. N-acetylcysteine (NAC) is a stable, safe and highly bioavailable antioxidant that shows promising prospects in bone tissue engineering due to the ability to attenuate oxidative stress and enhance the osteogenic potential and immune regulatory function of cells. This review systematically introduces the antioxidant mechanism of NAC, analyzes the advancements in NAC-related research involving mesenchymal stem cells, precursor cells, innate immune cells and animal models, discusses its function using the classic oral microenvironment as an example, and places particular emphasis on the innovative applications of NAC-modified tissue engineering biomaterials. Finally, current limitations and future prospects are proposed, with the aim of providing inspiration for targeted readers in the field.

## 1 Introduction

Bone defects are commonly encountered in clinical practice and can arise from various causes, including trauma (e.g., fractures), age-related bone loss (e.g., osteoporosis), infections (e.g., osteomyelitis and periodontitis), cancer (e.g., surgical resection), genetic diseases (e.g., inherited bone marrow failure syndrome), and congenital defects (e.g., cleft palate). The different manifestations of bone defects affect the quality of life to varying degrees. Bone tissue undergoes continuous remodeling, allowing for the restoration of structure and function after injury (Guo et al., 2021; Riquelme et al., 2021; Zhang et al., 2022). Small bone injuries (less than 6 mm in diameter) often heal spontaneously in a favorable microenvironment, while larger bone defects typically require surgical intervention and bone substitutes (Koushik et al., 2023). Approximately 2.2 million bone grafts are performed globally each year, ranking as the second most common tissue transplantation after blood transfusion (Sivakumar et al., 2022).

Autologous bone transplantation is currently considered the gold standard for bone defect restoration, distinguished by its osteogenesis, osteoconduction and osteoinduction properties. However, the application is limited by the scarcity of donor tissue, additional trauma to the patient and surgical complications (Hao et al., 2022; Sivakumar et al., 2022; Koushik et al., 2023). In contrast, alternative materials such as allografts and xenografts for bone defect repair carry potential risks including antigenicity, pathogen transmission, immune rejection and graft resorption (Sivakumar et al., 2022; Koushik et al., 2023). Bone tissue engineering has recently become a leading field in tissue engineering research, demonstrating promising therapeutic effects for bone defect repair by using seeding cells, osteoinductive factors, and biomaterial scaffolds. Stem cells, as primary seeding cells, play a crucial role in osteogenic differentiation within bone tissue engineering. The osteogenic function of seeding cells is intricately linked to the osteoimmune microenvironment where they reside (Takayanagi, 2007). Within the realm of bone tissue engineering, this microenvironment comprises both osteogenic and immune cell lineages, including mesenchymal stem cells (MSCs) and pre-osteoblasts as the principal osteogenic cells, and macrophages as the dominant immune cells (Li et al., 2024).

The complexity of the bone defect microenvironment makes cells vulnerable to various factors during regeneration and repair, creating substantial challenges for bone healing and clinical implementation of bone tissue engineering. Among these challenges, oxidative stress, driven by reactive oxygen species (ROS), is recognized as a major factor in cellular dysfunction (Sies and Jones, 2020; Li et al., 2021a; Bădilă et al., 2022; Sies et al., 2022). Research has shown that following bone injury, there is a marked increase in ROS levels, which peak during the healing phase and then progressively return to baseline values (Li J. et al., 2021). As the natural byproducts of cellular redox processes, ROS are typically neutralized by endogenous antioxidant systems. In oxidative environments such as *in vitro* expansion or *in vivo* transplantation, cells confront excessive ROS levels. This overexposure pattern depletes intracellular antioxidants, disrupts redox balance and leads to adverse effects, including cell apoptosis, proliferation inhibition, and functional impairment. Such consequences will ultimately hinder tissue regeneration and repair processes (Sies and Jones, 2020; Sies et al., 2022).

Exogenous antioxidants supplement the endogenous antioxidant system to counteract ROS, demonstrating beneficial outcomes in bone tissue engineering (Forman and Zhang, 2021; Sies et al., 2022). Glutathione (GSH), a critical endogenous antioxidant, is most effective when supplemented externally. However, its propensity for rapid oxidation and deactivation largely limits the bioavailability. Consequently, ongoing research explores more stable GSH precursor drugs (Forman and Zhang, 2021). N-Acetylcysteine (NAC), a small-molecule compound (C_5_H_9_NO_3_S), represents a major GSH precursor. Widely utilized in fundamental and clinical research, NAC features stable chemical properties, optimal safety and high bioavailability (Pedre et al., 2021). It has been currently used for the treatment of various pathologies, including cystic fibrosis, nephropathy and paracetamol toxicity. As a classical antioxidant, NAC produces potent antioxidative effects through direct and indirect pathways and is approved by regulatory agencies, including the Food and Drug Administration (FDA) of the United States and China (Rushworth and Megson, 2014; Pedre et al., 2021; Tenório et al., 2021). NAC has been investigated in over 500 clinical studies and is distinguished as the only antioxidant that enters phase IV clinical trials. The research related to NAC has maintained a steady publication rate of approximately 1,000 articles annually since 2011, which underscores its significance (Supplementary Figure S1A). Recent studies highlight the effectiveness of NAC in mitigating ROS, fostering pro-repairative microenvironment and enhancing the osteogenic potential and immune regulatory function of cells, which offers a promising role in the treatment of bone injuries and bone tissue engineering (Bădilă et al., 2022). As a result, NAC has attracted widespread interest in the field of stem cell biology and bone regenerative medicine (Supplementary Figure S1B). This article systematically introduces the antioxidant mechanism of NAC and its role in providing osteogenic microenvironment, summarizes its application in bone tissue engineering and the treatment of oral disease, and discusses the current challenges and future directions.

## 2 The antioxidant mechanisms of NAC

**FIGURE 1:**
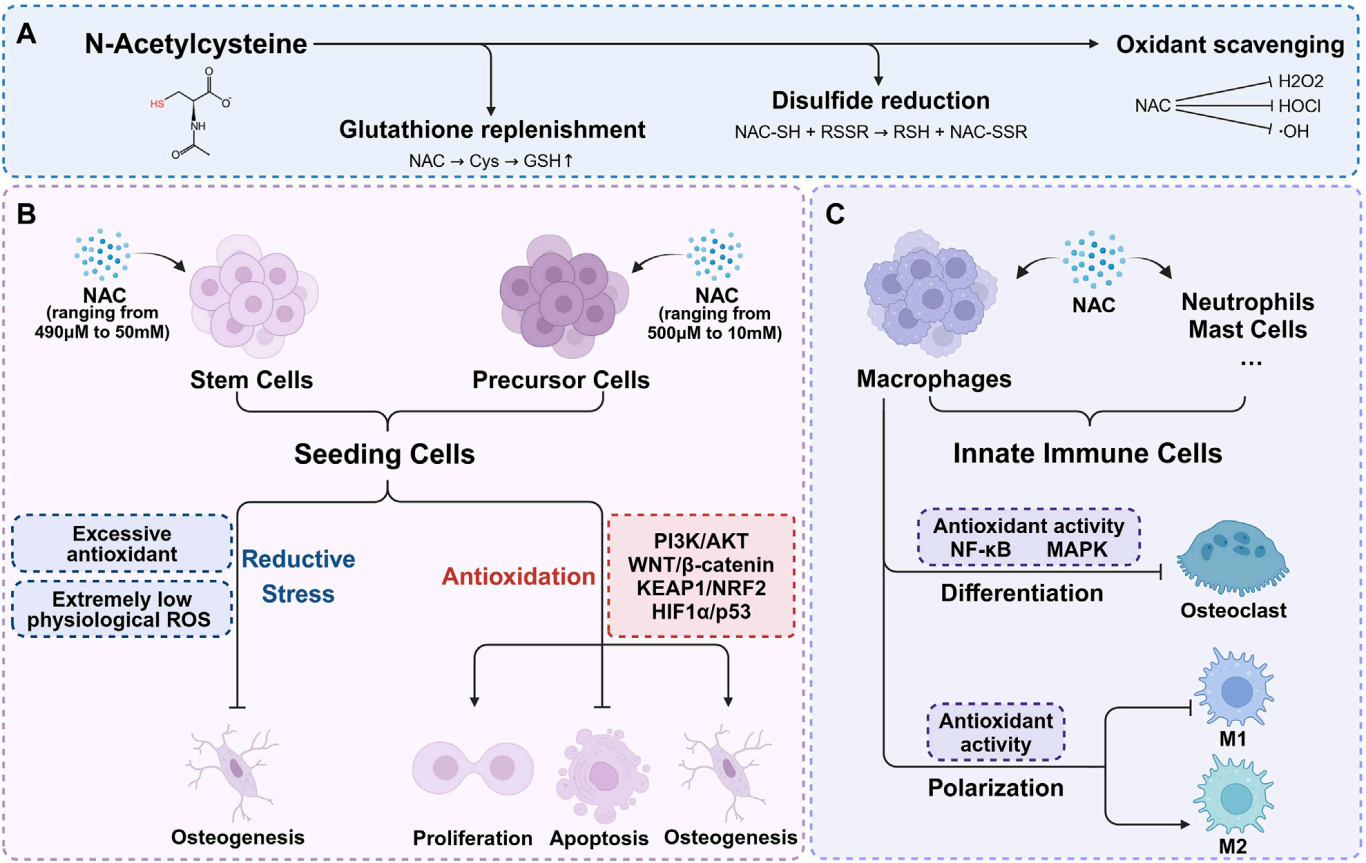
The antioxidant mechanisms of NAC and its role in bone microenvironment. **(A)** The antioxidant mechanisms of NAC are typically attributed to three main aspects: supplementing GSH, directly scavenging ROS, and reducing the disulfide bonds. **(B)** Within appropriate concentration range, NAC enhances seeding cells proliferation, inhibits apoptosis, and significantly promotes osteogenic differentiation. Higher concentration of NAC may result in osteogenic inhibition. **(C)** NAC regulates redox signaling pathways, mitigates oxidative stress, thereby inhibiting osteoclast differentiation and reducing bone resorption. With the exception of redox balance, NAC plays a part in macrophage polarization regulation.

The antioxidant mechanisms of NAC are typically attributed to three main aspects: supplementing GSH, directly scavenging ROS, and reducing the disulfide bonds (Figure 1A). While existing literature provides experimental evidence for these mechanisms, it often generalizes specific findings as universally applicable which leads to an incomplete or even biased understanding for the antioxidant action of NAC. The role of NAC in GSH supplementation has recently gained broader recognition. Although a few researches also support the effectiveness of NAC as a direct ROS scavenger and a disulfide bond reducing agent, further studies are necessary due to the lack of direct evidence.

### 2.1 GSH supplementation

GSH is synthesized and maintained at relatively high concentrations (∼mM) within cells, playing a vital role as an endogenous antioxidant. It can not only directly participate in redox reactions, but also act as a substrate or cofactor for numerous detoxifying enzymes (Meister and Anderson, 1983). As a cysteine precursor, NAC undergoes deacetylation to form cysteine which is a rate-limiting substrate for *de novo* GSH synthesis. Therefore, NAC can promote the synthesis of GSH and replenish the antioxidant system, and plays a role in GSH depletion conditions, such as acetaminophen or organophosphate poisoning (Abdel-Daim et al., 2019; Pettie et al., 2019; Turkmen et al., 2019). Additionally, NAC shows protective effects in chronic conditions marked by GSH depletion, including respiratory infections, cystic fibrosis, and diabetes (Asher and Guilford, 2016; Rosa et al., 2018; Guerini et al., 2022). As precursors for GSH synthesis used for treatment, NAC offers several advantages over direct cysteine supplementation. Firstly, NAC is safe even at high doses (exceeding 6 g/kg orally or 2 g/kg intravenously) (Bonanomi and Gazzaniga, 1980), while excessive cysteine intake can lead to severe pathological effects such as weight loss, cerebral damage, severe hypoglycemia, muscle spasms, and potentially fatal outcomes (EzEldeen et al., 2019). The biosafety of NAC comes from multiple aspects. NAC is more resistant to metal-catalyzed auto-oxidation which is the major source of cysteine toxicity (Wang and Cynader, 2001; Winterbourn et al., 2002). Moreover, cysteine is the sole endogenous source of hydrogen sulfide in mammals (Szabo, 2018). Direct supplementation of cysteine can induce rapid elevations in highly cytotoxic hydrogen sulfide levels. In contrast, NAC, bearing a negative charge at physiological pH, demonstrates slower cellular uptake due to its N-acetyl group, which hinders both passive and active membrane transport. Through deacetylation, NAC ensures a gradual increase in hydrogen sulfide levels within physiological limits, thereby facilitating cellular protection (Furne et al., 2008; Ezeriņa et al., 2018; Jurkowska and Wróbel, 2018; Faria et al., 2019). Secondly, freshly prepared NAC solutions exhibit greater resistance to air oxidation compared to cysteine solutions, with degradation rates at room temperature and under refrigeration of 0.89% and 0.48%, respectively (Siddiqui et al., 2016; Aldini et al., 2018). The oxidation of thiol groups in NAC requires deprotonation to form anions. Unlike cysteine, the acetylated amino group of NAC is unable to deprotonate and assume a positive charge, enhancing its oxidative resistance. Additionally, NAC maintains the solubility of its oxidation products, even in oxidized states (Pedre et al., 2021).

While NAC effectively replenishes GSH in GSH-deficient conditions, its ability to increase GSH levels under normal conditions is generally limited (Giustarini et al., 2012). Interestingly, exogenous NAC continues to provide benefits even when GSH synthesis is impaired (Nazıroğlu et al., 2013; Gleixner et al., 2017), indicating a GSH-independent protective mechanism. In addition to the direct antioxidant properties, there is evidence suggesting that NAC supplies sulfane sulfur to tissue cells. Sulfane sulfur refers to sulfur atoms with six valence electrons but no charge, which is actively involved in redox signaling. Sulfane sulfur enhances resistance to oxidative stress, protects cellular vitality and function through safeguarding protein thiol, stimulates protein activity and neutralizes free radicals (Pedre et al., 2021). Further research is needed to fully understand the functional mechanism of NAC independent from GSH replenishment.

### 2.2 Direct ROS scavenger

NAC is a thiol compound that exhibits a high propensity for reacting with oxidants from a chemical thermodynamic perspective. The chemical characteristics have led to numerous studies associating antioxidant mechanisms of NAC with the direct reaction with ROS. However, crucial factors include the reaction rate constants and the *in vivo* concentration of NAC (Jurkowska and Wróbel, 2018). In chemical reactions with primary ROS such as hydrogen peroxide (H_2_O_2_) and superoxide, the reaction rates of NAC are lower than those of endogenous antioxidants, including GSH, GSH peroxidase, and superoxide dismutase (Samuni et al., 2013). On the other hand, NAC shows stronger reactivity towards hypochlorite and nitrogen dioxide, partially explaining its direct antioxidant effects against exogenous factors like diet, air pollution, or certain inflammatory conditions (Meyer et al., 1994; Samuni et al., 2013). *In vivo* studies have shown that intravenously administered NAC reaches high concentrations in organs such as the lungs (320 μM), kidneys (250 μM), heart (170 μM), and liver (100 μM), with peak concentrations in human red blood cells reaching 200 μM. Although these concentrations are lower than GSH levels, they suggest that NAC possesses some degree of direct antioxidant activity (Medved et al., 1985; Giustarini et al., 2012; Pedre et al., 2021). Consequently, the role of NAC as a direct ROS scavenger, particularly against superoxide and hydrogen peroxide, to protect cells from oxidative damage, remains a topic of debate.

### 2.3 Disulfide bond reducing agent

NAC participates in a conventional sulfhydryl–disulfide exchange reaction, serving as an effective reducing agent for protein disulfide bonds. The reaction rate is influenced by the nucleophilicity of the thiolate, resulting in the enhanced capacity of NAC for reducing disulfide bonds compared to cysteine and GSH (Noszál et al., 2000). NAC is capable to disrupt disulfide bonds to alleviate cellular oxidative stress. This disulfide bonds reducing function can decrease the viscosity of glycoprotein, thereby reducing sputum viscosity, which explains the role of NAC as a mucolytic agent (Javitt et al., 2020). Additional evidence suggests that the reducing reaction of NAC can replenish small molecule thiols and regulate the redox state, as well as facilitate cysteine recycling through the formation of cystine or mixed disulfides involving cysteine (Aldini et al., 2018). Both pathways may contribute to antioxidant effects under the premise of higher NAC concentrations.

## 3 The role of NAC in bone microenvironment

### 3.1 NAC promotes the osteogenic differentiation of stem cells in the bone immune microenvironment

Among the MSCs sourced from various tissues, bone marrow-derived mesenchymal stem cells (BMSCs) and adipose-derived mesenchymal stem cells (AMSCs) are most widely used in bone tissue engineering (Hoang et al., 2022; Ju et al., 2023; Qin et al., 2023). Additionally, MSCs from dental tissues, such as periodontal ligament stem cells (PDLSCs), dental follicle stem cells (DFSCs) and stem cells from exfoliated deciduous teeth (SHEDs), demonstrate superior osteogenic differentiation and mineralization potential which are proven not to be inferior to BMSCs. They are also frequently obtained from routinely discarded human tissues, such as extracted third molars and deciduous teeth, making them highly accessible and raising minimal ethical concerns. Such merits make dental-derived MSCs have significant advantages in promoting bone tissue regeneration (Zheng et al., 2019; Srinivasan et al., 2021; Liu et al., 2022; Yan et al., 2022).

Varying concentrations of NAC ranging from 490 μM to 50 mM have been found to exhibit favorable cell compatibility in MSCs sourced from bone marrow, adipose and dental tissues. Within this concentration range, NAC not only preserves the activity of seeding cells or *in situ* MSCs through enhancing proliferation and inhibiting apoptosis, but also significantly promotes the progression of stem cell osteogenesis (Table 1). Extensive researches have focused on the antioxidant function of NAC and explored its positive impact on the osteogenic activity under stress conditions induced by exogenous stimuli such as H_2_O_2_ (Ueno et al., 2011; Yamada et al., 2019; Zhang J. et al., 2021; Liu et al., 2023), high glucose (Saito et al., 2022), ethanol (Chen et al., 2010), cyclic stress (Tan et al., 2015; Xi et al., 2022) and acrylonitrile (Sun et al., 2014). Additionally, primary BMSCs from castrated, aged, or transgenic osteoporosis mouse models (Xia et al., 2013; Yang et al., 2021; Shao et al., 2022) which experience deteriorating oxidative balance and increasing oxidative stress, are also used to assess the beneficial effects of NAC. The mechanism behind these effects is often attributed to the direct or indirect antioxidant activity of NAC and redox regulation of signal transduction, including WNT/β-catenin, TP53, and PI3K/AKT pathways. However, NAC demonstrates similar biological effects under normal culture conditions (Ji et al., 2011; Yamada et al., 2013; Debeljak Martacic et al., 2016; Yamada et al., 2019; Meng et al., 2022; Song et al., 2022). One explanation for this is the active sulfur provided by NAC, which metabolizes into hydrogen sulfide and sulfane sulfur. Both active sulfur compounds can regulate redox signals and promote osteogenic differentiation, thus contributing to bone homeostasis (Kowalczyk-Pachel et al., 2021; Gilbert et al., 2022). Another aspect to consider is the difference between *in vivo* and *in vitro* environments. Primary cells and immortalized cell lines are generally cultured under atmospheric oxygen levels, which are higher than the physiological oxygen content *in vivo*, leading to a pro-oxidative environment *in vitro* (Sies et al., 2022). To adapt to this environmental condition, cells depend on their redox system operating in reactive (feedback) and predictive (feedforward) modes, ultimately establishing new gene expression patterns which present as increased expression of antioxidant enzymes and stress defense proteins. This process is known as adaptive homeostasis or allostasis (Sies et al., 2022). The addition of exogenous antioxidants can help maintain the adaptive homeostasis. Thereby, NAC can exert antioxidant and pro-osteogenic effects *in vitro* without specific stimulus inducing oxidative stress.

**TABLE 1:** The application of NAC in MSCs.

Cell type	Species	NAC concentration	Induction	Function	Mechanism	Refs
BMSCs	Rat	5 mM	None, H_2_O_2_-induced oxidative stress	Restoring proliferation, inhibiting apoptosis, maintaining osteogenic differentiation, mineralization and expression of osteogenic factors	Antioxidant activity	Yamada et al. (2019)
		1 mM	High glucose-induced oxidative stress	Restoring proliferation, maintaining osteogenic differentiation and mineralization	Antioxidant activity	Saito et al. (2022)
		1 mM	Cyclic stress-induced oxidative stress	Maintaining osteogenic differentiation	Antioxidant activity	Tan et al. (2015)
		5 mM	H_2_O_2_-induced oxidative stress	Restoring proliferation, maintaining osteogenic differentiation, mineralization and expression of osteogenic factors	Antioxidant activity	Ueno et al. (2011)
		5 mM	None	Inhibiting apoptosis, promoting osteogenic differentiation, mineralization and expression of osteogenic factors	Osteogenic induction activity	Yamada et al. (2013)
		1 mM	Ethanol-induced oxidative stress	Maintaining osteogenic differentiation and expression of osteogenic factors	Activation of WNT/β-catenin pathway	Chen et al. (2010)
	Rabbit	490 μM	None	Promoting proliferation, osteogenic differentiation, mineralization and expression of osteogenic factors	Upregulation of WNT5A expression	Ji et al. (2011)
	Mouse	50 mM	Isolated from castrated osteoporosis mouse models	Maintaining mineralization	Inhibition of TP53INP2 degradation via antioxidant activity	Yang et al. (2021)
		500 μM	18-month-old elderly mice	Maintaining expression of osteogenic factors	Inhibition of the HIF1α/p53 pathway via antioxidant activity	Shao et al. (2022)
		Unkown	Isolated from Tg2576 transgenic mice expressing ubiquitinated APPswe (Alzheimer models with skeletal aging-like osteoporosis)	Maintaining osteogenic differentiation	Antioxidant activity	Xia et al. (2013)
ADSCs	Horse	Unknown	None	Promoting expression of osteogenic factors	Antioxidant activity	Song et al. (2022)
DFSCs	Human	5 mM	None	Promoting proliferation, osteogenic differentiation, mineralization and expression of osteogenic factors	Activation of PI3K/AKT pathway and antioxidant activity	Meng et al. (2022)
		5 mM	H_2_O_2_-induced oxidative stress	Restoring proliferation, maintaining osteogenic differentiation, mineralization and expression of osteogenic factors	Antioxidant activity	Zhang J. et al. (2021)
PDLSCs	Human	5 mM	Cyclic mechanical stress-induced oxidative stress	Maintaining osteogenic differentiation and expression of osteogenic factors	Antioxidant activity and downregulation of NRF2 expression	Xi et al. (2022)
		1 mM (NAC), 2 mM(Carbonized polymer dots synthesized by NAC)	H_2_O_2_-induced oxidative stress	Restoring proliferation, maintaining osteogenic differentiation and mineralization	Antioxidant activity	Liu et al. (2023)
SHEDs	Human	1 mM	None	Promoting proliferation, osteogenic differentiation and mineralization	Antioxidant activity	Debeljak Martacic et al. (2016)
Umbilical cord MSCs	Human	3 mM	Acrylonitrile- induced oxidative stress	Restoring proliferation, inhibiting apoptosis and promoting osteogenic differentiation	Not mentioned	Sun et al. (2014)

### 3.2 NAC enhances the osteogenic activity of precursor cells in the bone immune microenvironment

Primary MSCs closely reflect the biological behavior of niche cells and are both ideal seeding cells and cell models in bone tissue engineering. However, these stem cells face challenges, including limited availability, significant heterogeneity, *in vitro* instability due to culture conditions as well as rising ethical concerns. Additionally, *in vitro* models derived from different species, such as rats and rabbits, complicate the extrapolation of results to human clinical conditions due to interspecies differences. Pre-osteoblastic cell lines with MC3T3-E1 as a prominent example, offer a more uniform and stable population of immortalized cells suitable for studies of bone tissue engineering (Czekanska et al., 2012). While they cannot fully substitute primary MSCs, they are recognized as valuable tools in the development of novel biomaterials and therapies.

Although not as prominently as in the case of primary stem cells, pre-osteoblastic cell lines such as MC3T3 and NH3T3 possess a certain degree of osteogenic capacity which can be regulated by the redox environment. Thereby, As shown in Figure 1B, NAC also exert pro-osteogenic effect through the antioxidative activity in pre-osteoblastic cells under stress conditions induced by H_2_O_2_ (Lee et al., 2015), high glucose (Liu and Yang, 2016), lipopolysaccharides (LPS) (Chu et al., 2023; Li et al., 2023), 7-ketocholesterol (7KC) (Ouyang et al., 2022), dibutyl phthalate (DBP) (Cui et al., 2022), TNF-α (Zhang et al., 2017), dexamethasone (Deng et al., 2019), MT3 knockout (Li S. et al., 2021) and adenosine triphosphate (ATP) (Chu et al., 2023) (Table 2). Similar to those in MSCs, the concentrations of NAC used in precursor cells range from 500 μM to 10 mM. While NAC promotes osteogenesis across a broad concentration range, the effectiveness does not always positively correlate with concentration. Applying higher concentrations of NAC to stem cells or precursor cells may result in osteogenic inhibition (Arakaki et al., 2013; Meng et al., 2022), potentially due to excessive antioxidant-induced reductive stress. The imbalance in cellular redox homeostasis characterized by a disruption between oxidants and antioxidants, is a defining feature of various pathological states. Elevated ROS levels lead to antioxidant depletion, causing oxidative stress that impairs stem cell functionality. However, physiological levels of ROS and their associated redox signaling networks are equally vital for maintaining cellular function and redox balance. Excessive antioxidants can inhibit the accumulation of physiological ROS necessary for signal transduction in stem cells, leading to reductive stress and consequently hampering their biological behavior (Manford et al., 2020; Coombs et al., 2021; Manford et al., 2021; Sies et al., 2022). The precise impact of oxidant and antioxidant levels on different cell types, and the necessity of maintaining these levels within a specific range, remains unclear due to current research limitations (Sies and Jones, 2020). It suggests that the appropriate regulation of redox homeostasis is a critical consideration and is instructive to the clinical application of NAC.

**TABLE 2:** The application of NAC in precursor cells.

Cell type	Species	NAC concentration	Induction	Function	Mechanism	Refs
MC3T3-E1	Mouse	5 mM	H_2_O_2_-induced oxidative stress	Maintaining osteogenic differentiation, mineralization and expression of osteogenic factors	Inhibition of NRF2/HO-1 pathway	Lee et al. (2015)
		Unknown	High glucose-induced oxidative stress	Restoring proliferation, maintaining osteogenic differentiation and mineralization and	Inhibition of PI3K/AKT pathway via antioxidant activity	Liu and Yang (2016)
		500 μM	LPS-induced oxidative stress	Restoring proliferation, maintaining osteogenic differentiation, mineralization and expression of osteogenic factors	Antioxidant activity	Li et al. (2023)
		2.5 mM	7KC-induced oxidative stress	Inhibiting apoptosis, maintaining osteogenic differentiation, mineralization and expression of osteogenic factors	Inhibition of autophagy via antioxidant activity	Ouyang et al. (2022)
		5 mM	DBP-induced oxidative stress	Inhibiting apoptosis, maintaining expression of osteogenic factors	Inhibition of mitophagy via antioxidant activity	Cui et al. (2022)
		1 mM	TNF-α-induced inflammatory stimulation	Restoring proliferation, maintaining osteogenic differentiation and expression of osteogenic factors	Downregulation of DRP1 expression via antioxidant activity	Zhang et al. (2017)
		500 μM	Dexamethasone-induced oxidative stress	Restoring proliferation, inhibiting apoptosis, maintaining osteogenic differentiation	Activation of PI3K/AKT/GSK3β pathway via antioxidant activity	Deng et al. (2019)
		10 mM	None	Inhibiting mineralization	Reductive stress	Arakaki et al. (2013)
C2C12	Mouse	10 mM	MT3 knockout-induced oxidative stress	Maintaining osteogenic differentiation and expression of osteogenic factors	Antioxidant activity	Li S. et al. (2021)
Periodontal ligament fibroblasts	Human	10 mM	LPS/ATP-induced pyroptosis	Restoring proliferation, maintaining osteogenic differentiation, mineralization and expression of osteogenic factors	Antioxidant activity and inhibition of SIRT1/NF-κB/Caspase-1 pathway-induced pyroptosis	Chu et al. (2023)

While these findings highlight the multifaceted benefits of NAC, it is imperative to note that the current body of research may not fully encapsulate the complex role of NAC in osteogenesis by regulating the stem cells and precursor cells in bone microenvironment. Despite the promising results, there remains a need for more comprehensive studies that critically evaluate the underlying mechanisms and potential clinical implications. For instance, the variability in the efficacy of NAC across different studies suggests that the context of its application, including the specific stress conditions and cell types involved, plays a crucial role in determining its effectiveness. Thus, future research should aim to delineate these contextual factors more clearly, providing a more nuanced understanding of how NAC can be optimally leveraged for therapeutic purposes in bone regeneration.

### 3.3 NAC regulates innate immune cells in the bone immune microenvironment to reduce bone resorption

In bone tissue engineering, the osteogenic potential of seeding cells including MSCs and precursor cells, is intricately linked to their immune microenvironment. Therefore, the process of bone regeneration is significantly influenced by the stimulatory role of the immune system on osteogenesis (Mensah et al., 2009). The interdisciplinary field “osteoimmunology” which was first coined by Arron and Choi explores the interplay between the skeletal and immune systems (Arron and Choi, 2000). Hematopoietic stem cells, originating in the bone marrow, can differentiate into all types of cells of the mammalian immune system. Both bone and immune cells coexist within the same microenvironment and are regulated by various common factors, collaboratively contributing to the functions of the bone-immune system (Walsh et al., 2006; Takayanagi, 2007). Notably, the immune system influences bone metabolism primarily through the innate and adaptive response. Among the innate components, innate immune cells, such as macrophages, neutrophils and mast cells, are capable of producing a range of cytokines that regulate bone metabolism in skeletal diseases (Walsh et al., 2018; Tsukasaki and Takayanagi, 2019). The crucial bone-resorbing function of osteoclasts which originate from macrophages, also highlight the significant contribution of innate immune cells to the bone-immune system (Edwards and Mundy, 2011). As a result, modulation of the innate immune cells is a feasible way to foster favorable conditions for bone regeneration in bone tissue engineering.

NAC possesses immunomodulatory properties, notably by inhibiting oxidation-sensitive signaling pathways such as NF-κB and MAPK (Tieu et al., 2023). The focus of current research on the role of NAC in bone metabolism regulation via innate immunity primarily centers on macrophage modulation, with fewer studies addressing its effects on neutrophils, mast cells and other innate immune cells (Figure 1C). For instance, 8 mM NAC has been shown to partially inhibit M1 macrophage polarization and restore M2 macrophage polarization in diabetic periodontitis patients by scavenging ROS. The change of polarization can modulate the production of proinflammatory and anti-inflammatory cytokines by macrophages, thus regulating the bone-immune microenvironment and preventing alveolar bone loss (Pajarinen et al., 2019; Zhang B. et al., 2021). Conversely, other research reveals that sustained low ROS levels may enhance M2 macrophage polarization without significantly affecting M1 macrophages (Qiu et al., 2023). Further investigation is required to clarify the role of NAC in macrophage polarization regulation. Additionally, —NAC has been observed to scavenge ROS in bone marrow-derived macrophages, RAW264.7 cells, and CD14+ peripheral blood monocytes under conditions promoting osteoclast differentiation induced by RANKL, M-CSF (Aitken et al., 2004; Lee et al., 2005; Sakai et al., 2012; Cao and Picklo, 2014; Kim et al., 2018; Soares et al., 2019; Guo et al., 2020), H_2_O_2_ (Liu et al., 2021), Trimethylamine-N-oxide (TMAO) (Wang et al., 2022), LPS (Yan et al., 2020), ferric ammonium citrate (FAC) (Jia et al., 2012), TRP14 knockout (Hong et al., 2014), and NRF2 knockout (Hyeon et al., 2013; Yang et al., 2023). By these manners, NAC can mitigate ROS and inhibit osteoclast differentiation via regulating redox signaling pathways, thereby reducing oxidative stress-mediated bone resorption (Table 3). Although there is a certain amount of *in vitro* studies investigating the effect of NAC on innate immune cells, it has just begun to be explored *in vivo*. Therefore, a deeper understanding of the regulatory and signaling mechanisms of NAC on macrophages and other innate immune cells involved in bone metabolism will further inform its application in bone tissue engineering. What’s more, While the immunomodulatory properties of NAC are well-documented, its precise mechanisms in many cellular contexts still remain underexplored. The conflicting findings regarding M1 and M2 macrophage polarization suggest that the effects of NAC may be context-dependent, influenced by factors such as concentration, cell type, and specific microenvironmental conditions. This highlights a critical gap in current research, where a more nuanced approach is necessary to illustrate the circumstances under which NAC exerts beneficial effects. Future studies should focus not only on the antioxidative and immunomodulatory effects of NAC, but also on critically assessing how these properties translate to *in vivo* systems. This will provide a clearer understanding of the potential therapeutic applications of NAC and guide its effective use in clinical settings.

**TABLE 3:** The application of NAC in innate immune cells.

Cell type	NAC concentration	Induction	Function	Mechanism	Refs
BMDM	30 mM	M-CSF, RANKL, TMAO	Inhibiting osteoclast differentiation	Antioxidant activity and inhibition of NF-κB pathway	Wang et al. (2022)
	20 mM	M-CSF, RANKL, LPS	Inhibiting osteoclast differentiation, reducing bone resorption	Antioxidant activity	Yan et al. (2020)
	Unkown	M-CSF, RANKL	Inhibiting osteoclast differentiation, suppressing endoplasmic reticulum stress and autophagy	Antioxidant activity and downregulation of PERK expression	Guo et al. (2020)
	200 nM	M-CSF, RANKL	Inhibiting osteoclast differentiation	Antioxidant activity and inhibition of TPC2 calcium channel	Soares et al. (2019)
	Unkown	M-CSF, RANKL	Inhibiting osteoclast differentiation	Antioxidant activity and inhibition of endoplasmic reticulum stress and CREBH/NFATc1 signaling axis	Kim et al. (2018)
	10 mM	M-CSF, RANKL, NRF2 knockout	Inhibiting osteoclast differentiation	Antioxidant activity	Hyeon et al. (2013)
	10 mM	RANKL	Inhibiting osteoclast differentiation	Antioxidant activity, downregulation of OH-1 and inhibition of HMGB1 release and Caspase-3-dependent pathway	Sakai et al. (2012)
	30 mM	RANKL	Inhibiting osteoclast differentiation	Antioxidant activity and inhibition of MAPK pathway	Lee et al. (2005)
RAW264.7	100 μM	RANKL, H_2_O_2_	Inhibiting osteoclast differentiation, reducing bone resorption	Antioxidant activity and inhibition of NF-κB pathway	Liu et al. (2021)
	4 mM	M-CSF, RANKL, NRF2 knockout	Inhibiting osteoclast differentiation	Antioxidant activity	Yang et al. (2023)
	20 mM	RANKL	Inhibiting osteoclast differentiation, reducing bone resorption	Antioxidant activity	Cao and Picklo (2014)
	5 mM	RANKL, TRP14 knockout	Inhibiting osteoclast differentiation	Antioxidant activity and inhibition of NF-κB and MAPK pathway	Hong et al. (2014)
	10 mM	RANKL, FAC	Inhibiting osteoclast differentiation, reducing bone resorption	Antioxidant activity	Jia et al. (2012)
CD14+ peripheral blood monocytes	10 mM	M-CSF, RANKL	Inhibiting osteoclast differentiation	Antioxidant activity, upregulation of TBP-1, inhibition of TRX-1 nuclear translocation and activation of AP-1 and NF-κB	Aitken et al. (2004)

### 3.4 NAC promote bone tissue regeneration in animal models

The osteogenic potential of NAC has been substantiated through animal experiments (Table 4). Bone defect models are commonly used in such studies. When loaded on scaffolds such as collagen sponges and treated dentin matrix (TDM) and subsequently implanted into bone defects, NAC or NAC-pretreated MSCs can significantly enhance collagen deposition, bone formation rate, bone volume/total volume (BV/TV), bone mineral density (BMD) and trabecular bone parameters (thickness, number, separation). Moreover, this approach improves the survival rate of seeding cells via enhancing cell proliferation and/or inhibiting apoptosis (Yamada et al., 2013; Watanabe et al., 2018; Yamada et al., 2019; Zhang J. et al., 2021; Meng et al., 2022). In various bone defect-related models including diabetes, periodontitis and orthodontic tooth movement, NAC administration via injection has been shown to reduce bone loss, accelerate bone integration with implants and enhance bone remodeling on the tension side. Strategies such as oral gavage or addition to drinking water have also been effective in restoring *in vivo* osteogenic activity as well as in improving the mechanical properties of bones in diet-induced models and genetic models that simulating osteoporosis (Chen et al., 2010; Chen et al., 2011; Xia et al., 2013; Ma J. et al., 2022). Unfortunately, the therapeutic potential of NAC in bone tissue pathology has not been substantiated by clinical trials (https://clinicaltrials.gov/), underscoring the need for further *in vivo* research.

**TABLE 4:** The application of NAC in bone defect-related animal models.

Animal model	Application form	Administration strategy	Function	Mechanism	Refs
Bone defect model					
Rat femoral defect model	Collagen sponge containing 5 mM NAC	*In situ* transplantation	Improving BV/TV and trabecular bone parameters	Osteogenic induction activity	Yamada et al. (2013)
Rat femoral defect model	Collagen sponge containing BMSCs pre-treated with 5 mM NAC	*In situ* transplantation	Improving BV/TV and trabecular bone parameters	Antioxidant activity	Yamada et al. (2019)
Rat femoral defect model	Collagen sponge containing BMSCs pre-treated with 5 mM NAC	*In situ* transplantation	Inhibiting apoptosis of seeding cells, improving of BV/TV and BMD	Antioxidant activity	Watanabe et al. (2018)
Rat mandibular defect model	TDM biological tooth root composites containing DFSCs pre-treated with 5 mM NAC	*In situ* transplantation	Inhibiting apoptosis of seeding cells, improving of collagen deposition	Antioxidant activity	Zhang J. et al. (2021)
Rat alveolar bone defect model	Collagen sponge containing 5 mM NAC or BMSCs pre-treated with 5 mM NAC	*In situ* transplantation	Improving BV/TV and BMD	Activation of PI3K/AKT pathway and antioxidant activity	Meng et al. (2022)
Closed fracture model in rats with ethanol diet	NAC solution at a dose of 200 mg/kg body weight	Intraperitoneal injection, once daily for three consecutive days	Enhancing bending strength and expression of osteogenic factors	Antioxidant activity	Duryee et al. (2018)
Disease models					
Diabetic sheep model	NAC solution at a dose of 5 mg/kg body weight	Intramuscular injection, once weekly for 12 consecutive weeks	Improving BV/TV and osseointegration rate of titanium alloy implants	Activation of FAK/BMP/SMAD pathway via antioxidant activity	Ma et al. (2017)
Diabetic rabbit model	NAC solution at a dose of 5 mg/kg body weight	Intravenous injection, once weekly for 10 consecutive weeks	Improving BV/TV and osseointegration rate of titanium alloy implants	Activation of WNT/β-catenin pathway via antioxidant activity	Ma et al. (2018)
Diabetic rabbit model	NAC solution at a dose of 5 mg/kg body weight	Intravenous injection, once weekly for 10 consecutive weeks	Improving BV/TV and osseointegration rate of titanium alloy implants	Activation of PI3K/AKT pathway via antioxidant activity	Ma X. et al. (2022)
Periodontitis mouse model	NAC solution at a dose of 100 mg/kg body weight or Carbonized polymer dot solution	Intraperitoneal injection, once daily for four consecutive weeks	Reducing alveolar bone resorption, improve collagen deposition, BV/TV and expression of osteogenic factors	Inhibition of KEAP1, activate NRF2 and antioxidant activity	Liu et al. (2023)
Gene regulation models					
Hepcidin knockout mouse model	NAC solution at a dose of 100 mg/kg body weight	Oral gavage, administered at weekly intervals three times, for a consecutive four weeks	Improving bone formation rate, BV/TV, BMD, trabecular bone parameters, mechanical strength, and expression of osteogenic factors	Antioxidant activity	Ma J. et al. (2022)
Transgenic mouse model of Alzheimer’s disease (Tg2576) expressing ubiquitinated APPswe	NAC solution at a dose of 2 mg/kg body weight	Oral administration via drinking water, for four consecutive weeks	Improving collagen deposition, BV/TV and trabecular bone parameters	Antioxidant activity	Xia et al. (2013)
Other Models					
Orthodontic tooth movement rat model	NAC solution at a dose of 225 mg/kg body weight	Intraperitoneal injection, once daily for 2–4 consecutive weeks	Improving BV/TV, trabecular bone parameters, structural model index, and expression of osteogenic factors on the tension side	Antioxidant activity and downregulation of NRF2 expression	Xi et al. (2022)
Lactation ethanol diet rat model	NAC solution at a dose of 1.4 g/kg body weight	Oral administration via drinking water, for four consecutive weeks	Improving BV/TV and expression of osteogenic factors	Activation of WNT/β-catenin pathway	Chen et al. (2010)
Ethanol diet cycling rat model	NAC solution at a dose of 1.2 g/kg body weight	Oral administration via drinking water, for four consecutive weeks	Improving BV/TV and BMD, increasing osteoblast count, osteoblast surface ratio, and decreasing eroded surface ratio	Inhibition of NADH oxidase	Chen et al. (2011)

Similar to the *in vitro* experiments, NAC primarily influences osteogenic effects through ROS and related redox signaling pathways in animal models. The classical redox pathways including WNT/β-catenin, PI3K/AKT, NRF2, and p53 can respond to oxidant or antioxidant signals and significantly regulate gene transcription (Holmström and Finkel, 2014; Lennicke and Cochemé, 2021). NAC has been shown to activate the WNT pathway in various cell and animal models (Chen et al., 2010; Ji et al., 2011; Ma et al., 2018), but its effects on PI3K/AKT, NRF2, and other pathways are not consistent. For example, in MC3T3-E1 cells cultured in high glucose, NAC inhibits the PI3K/AKT pathway (Liu and Yang, 2016), while in dexamethasone-stimulated MC3T3-E1 cells and human DFSCs cultured under standard conditions, it activates PI3K signaling (Deng et al., 2019; Meng et al., 2022). This variability may stem from the complex feedback networks involving ROS-mediated redox and phosphorylation modifications (Su et al., 2019; Gou et al., 2022; Kuznetsov et al., 2022). Redox-dependent modifications of numerous intermediates can lead to diverse functional results such as changes in activity, localization and substrate specificity. In addition, oxidation of substrates may alter their localization/binding partners and/or their cognate phosphatase activity, further exacerbating signal dysregulation. While the *in vivo* evidence supporting the osteogenic potential of NAC is promising, the inconsistency in its effects across different signaling pathways suggests a research gap. The variability in the influence of NAC on pathways like PI3K/AKT suggests that its efficacy may be highly context-dependent. This underscores the importance of not only continuing to explore the antioxidative and osteogenic effects of NAC, but also critically examining the underlying mechanisms in varied biological contexts. Furthermore, the lack of clinical trials evaluating the therapeutic potential of NAC in bone pathology emphasizes the need for translational research that bridges the gap between preclinical findings and clinical applications. By focusing on these areas, future studies can provide a more comprehensive understanding of how NAC can be effectively utilized in tissue engineering and regenerative medicine.

## 4 NAC provides a pro-osteogenic microenvironment for alveolar bone repair

Alveolar bone is the most metabolically active bone in the skeletal system that is under active bone remodeling. Due to the continuous exposure to occlusal forces and periodontal microorganisms, alveolar bone possesses a unique immune microenvironment distinct from the long bones, with more frequent interactions between monocytes/macrophages and MSCs (Lin et al., 2021). The excessive production of ROS has been detected in many settings of alveolar bone injuries, including periodontitis, implant osseointegration and orthodontics-related bone loss. Alveolar bone defects are important and major issues in clinical work that pose considerable challenges for subsequent implant and restorative treatments. These defects arise from multiple factors, with periodontitis being the most extensively studied (Kinane et al., 2017). Periodontitis results from an overactive immune response to periodontal pathogen (Sima et al., 2019). Immune cells produce excessive ROS and lead to an oxidative stress microenvironment while killing pathogenic bacteria. The oxidative stress microenvironment subsequently inhibits osteogenic differentiation and bone formation, which is the major contributor to periodontal tissue defects (Liao et al., 2016; Liu et al., 2017). NAC has shown therapeutic promise when used alone or in combination with stem-cell based bone tissue engineering for alveolar bone defects caused by periodontitis. NAC can provide a favorable osteoimmune microenvironment for osteogenic differentiation. On the one hand, NAC and its derivatives have been proven capable of sustaining physiological ROS level and restoring proliferation and osteogenic differentiation of PDLSCs (Qiu et al., 2021; Xi et al., 2022; Liu et al., 2023), which are considered the most promising endogenous MSCs for alveolar bone regeneration (Tsumanuma et al., 2011). Furthermore, as previously mentioned, NAC can also be applied in bone tissue engineering by stimulating osteogenic differentiation and mineralization potential of various seeding cells, thereby achieving better promotion of alveolar bone regeneration. On the other hand, NAC can suppress osteoclastogenesis by reducing ROS level in LPS-induced inflammatory microenvironment, consequently attenuating osteolysis (Yan et al., 2020). Overall, NAC can facilitate alveolar bone regeneration by promoting bone formation and inhibiting bone resorption, thus enabling a positive therapeutic effect for periodontal tissue defects caused by periodontitis.

Apart from the application in the treatment for periodontitis-related bone defects, NAC can also exert osteogenic function in dental implantology and orthodontics. The clinical success of dental implants primarily depend on the direct structural and functional connection between the living bone and the implant surface, which is referred to as osseointegration (Guglielmotti et al., 2019). A balanced oxidant level in the surrounding tissue is critical for implant osseointegration, preventing peri-implant infections, and enhancing the implant success rate (Mouthuy et al., 2016). Research indicates that surface treatment of implants with appropriate concentration of NAC can mitigate oxidative stress and reduce the upregulated expression of pro-inflammatory cytokines induced by LPS and hyperlipidemia. Such treatment enhances osteoblast adhesion and proliferation, thereby improving the biocompatibility and osseointegration of implants (Lee et al., 2013; Wang et al., 2021a; Li et al., 2023). Additionally, alveolar bone remodeling on the tension side during orthodontic treatment is highly dependent on the osteogenic differentiation of PDLSCs induced by cyclic mechanical stress. NAC can promote osteogenic differentiation of PDLSCs and facilitate bone formation on the tension side by reducing the excessive ROS generated by cyclic stress, enabling NAC to potentially improve bone remodeling and reduce adverse effects of orthodontics (Xi et al., 2022). In summary, although not yet applied clinically, NAC is capable of providing a favorable osteogenic microenvironment and has broad application prospects in periodontal, implant and orthodontic treatment.

## 5 NAC combined with biomaterials for bone tissue engineering

Bone tissue, a highly organized natural composite material, integrates organic and inorganic substances with various cell types within the extracellular matrix (ECM) scaffold (Koons et al., 2020). In an effort to mimic the microstructure of bone ECM, a diverse range of materials and combinations are employed as potential biomaterials for bone tissue engineering. The ideal biomaterials should provide mechanical support, a conducive microenvironment, and serve as carriers for bioactive molecules such as NAC during tissue regeneration. This aids in facilitating cell adhesion, proliferation, and differentiation (Koushik et al., 2023). At present, commonly used biomaterials include polymers, bioceramics, and composite materials. Given the composite nature of bone tissue and the intricate requirements of bone tissue engineering materials, composite materials have become the preferred choice, offering superior performance compared to single-component materials (Koons et al., 2020; Hassani et al., 2022).

**FIGURE 2:**
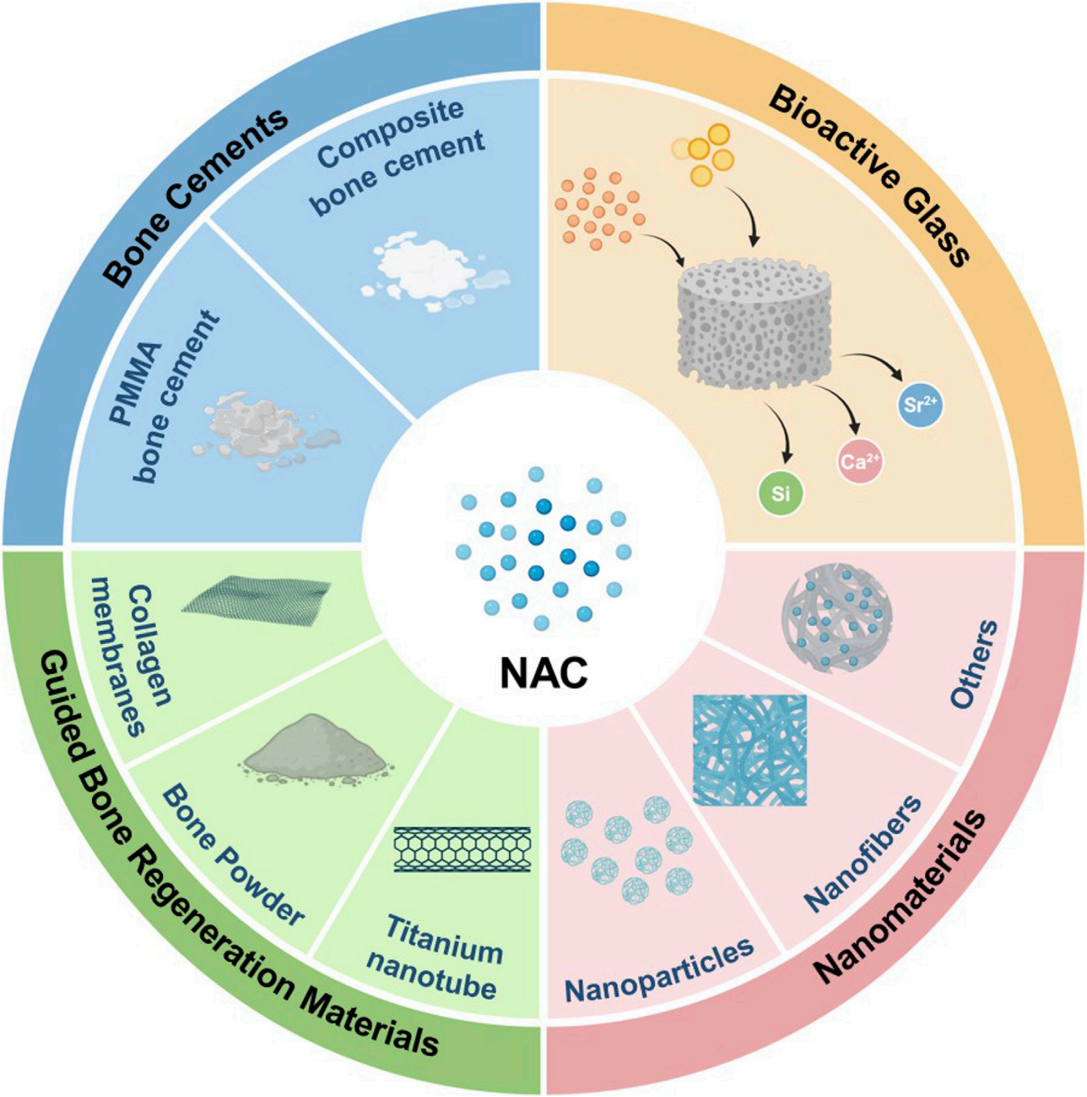
The application of NAC in bone tissue engineering.

Polymethyl methacrylate (PMMA) bone cement is extensively used in orthopedic surgery, but the high elastic modulus and low biocompatibility have limited further advancements (Zhu et al., 2020; Wang et al., 2021b). Incorporating NAC into PMMA bone cement has significantly improved its bioactivity, as evidenced by enhanced osteogenic activity in rat BMSCs and MC3T3-E1 cells and favorable outcomes in bone regeneration in a rat femoral defect model (Tsukimura et al., 2009; Zhao et al., 2019). Similarly, adding NAC to silk fibroin/tri-calcium phosphate composite bone cement has shown comparable osteogenic effects in both *in vitro* and *in vivo* experiments (Feng et al., 2020). In composite materials, bioactive glass and nanomaterials are common fillers (Koons et al., 2020). Bioactive glass, a significant bioceramic, releases ions like calcium, silicon, and strontium. Mesoporous bioactive glass with the structured mesoporous architecture enables the incorporation of various drugs and biomolecules, making it an innovative material in bone tissue engineering (Vallet-Regi and Salinas, 2021). The combination of NAC with strontium-doped mesoporous bioactive glass and thermosensitive polyurethane hydrogel efficiently enhances osteogenic factor expression in precursor cells (Pontremoli et al., 2022). Furthermore, NAC can be integrated into nanoparticles, nanofibers, and other nanomaterials to create nano-engineered composite materials. These materials facilitate osteogenic differentiation and mineralization of MSCs and have shown pro-osteogenic effects in periodontitis and calvarial defect models (Lee et al., 2018; Zhu et al., 2019; Li et al., 2021b; Qiu et al., 2021). Additionally, guided bone regeneration materials in oral and maxillofacial surgery such as resorbable collagen membranes and demineralized freeze-dried bone powders as well as titanium nanotube implants, can also be used as bone scaffolds for the delivery of NAC (Yamada et al., 2011; Lee et al., 2013). Despite that bioactive materials have obtained the positive results of bone defect repair, their clinical application faces challenges due to the scarcity of animal studies and unclear specific mechanisms in the research. While not yet translated into clinical practice, NAC presents significant potential for modifying various biomaterials and offers promising prospects in bone tissue engineering as an active molecule (Table 5 and Figure 2). Comprehensive animal studies and well-designed clinical trials will be essential to transition these innovative materials from bench to bedside.

**TABLE 5:** The application of NAC in tissue engineering biomaterials.

Carrier	*In vitro* studies	*In vivo* studies	Mechanism	Refs
PMMA bone cement	Inhibiting apoptosis, promoting proliferation, osteogenic differentiation, mineralization and expression of osteogenic factors in rat BMSCs	Improving bone contact area, BV/TV, mechanical strength in rat femoral defect models	Antioxidant activity	Tsukimura et al. (2009)
PMMA bone cement	Promoting mineralization in MC3T3-E1	None	Not mentioned	Zhao et al. (2019)
Silk fibroin/α-TCP bone cement	Promoting osteogenic differentiation, mineralization and expression of osteogenic factors in rat BMSCs	Improving BV/TV in rat femoral defect models	Activation of WNT/β-catenin pathway	Feng et al. (2020)
Strontium-doped mesoporous bioactive glass-modified thermo-sensitive polyurethane hydrogel	Promoting expression of osteogenic factors in human SAOS-2	None	Not mentioned	Pontremoli et al. (2022)
Enzyme-crosslinked gelatin/functionalized gold nanoparticle-modified hydrogel	Promoting proliferation and osteogenic differentiation in human ADSCs	None	Antioxidant activity	Lee et al. (2018)
Mesoporous silica nanoparticle-modified PLGA electrospinning system	Promoting proliferation, mineralization and expression of osteogenic factors in rat BMSCs	None	Not mentioned	Zhu et al. (2019)
Hydroxyapatite/silk fibroin biomimetic nanofibers	Promoting proliferation, osteogenic differentiation and expression of osteogenic factors in induced pluripotent stem cell-derived MSCs	Improving BV/TV and BMD in rat calvarial defect models	Not mentioned	Li et al. (2021b)
PEG-ss-PCL nanoparticle drug delivery platform	Inhibiting apoptosis, maintaining osteogenic differentiation, mineralization, and expression of osteogenic factors in LPS-stimulated human PDLSCs	Reducing alveolar bone resorption, improving collagen deposition in rat periodontitis models	Antioxidant activity	Qiu et al. (2021)
Bovine resorbable collagen membrane and human demineralized freeze-dried bone powder	Inhibiting apoptosis, promoting proliferation and osteogenic differentiation in rat osteoblasts	None	Antioxidant activity	Yamada et al. (2011)
Titanium nanotube implant	Promoting proliferation and expression of osteogenic factors in MC3T3-E1	Improving BV/TV, BMD and expression of osteogenic factors in rat mandibular first molar implant models	Antioxidant activity	Lee et al. (2013)

## 6 Conclusion and future perspectives

Therapeutic approaches to address oxidative stress typically involve upregulating antioxidant signals such as NRF2, administering exogenous antioxidants, supplementing trace elements or nutrients, and implementing environmental interventions (Forman and Zhang, 2021). As a precursor of GSH, NAC not only facilitates *de novo* GSH synthesis but also exhibits independent antioxidant effects. Despite the development of derivatives like N-acetylcysteine amide and N-acetylcysteine ethyl ester which offer improved cell uptake efficiency, bioavailability and antioxidant performance compared to NAC, their application potential for bone tissue engineering warrant further exploration due to the reduced oxidative stability and limited ability to counteract cysteine toxicity (Tosi et al., 2021; Eligini et al., 2023). Thus, NAC remains an indispensable antioxidant that acts as a pivotal strategy against oxidative stress in the treatment of oral disease and bone tissue engineering.

This review undertook a comprehensive literature search encompassing the areas of NAC and bone tissue engineering. It systematically categorized and analyzed the advancements in NAC research involving MSCs, precursor cells, innate immune cells and animal models, with a particular emphasis on the innovative applications of NAC-modified tissue engineering biomaterials. The review consolidates experimental evidence that supports the utilization of NAC in bone tissue engineering, underscoring its capacity to improve the osteogenic microenvironment and optimize bone regeneration outcomes. However, there are several major limitations to the current studies that require further investigation:

1. The research on immune microenvironment is insufficient, as it neither adopts a wider variety of immune cell types nor investigates the interactions between immune cells and osteoprogenitors, which hinders NAC to obtain an ideal application effect.
2. With the advancement of biomaterials, the optimal carrier for NAC in bone tissue engineering which possess the capacity of good mechanical strength, stable chemical characteristics and ideal loading and controlled release behaviors etc., remains to be further investigated.
3. Most of the studies focus on the effect of NAC on cells and animal models with a notable absence of comprehensive clinical trial data which would lead to unknown biological effects and human health risks, thereby impeding its clinical application.
4. The prevailing focus regarding oxidative stress often neglects reductive stress within the context of redox imbalances, potentially obscuring the adverse effects of NAC. The lack of in-depth investigation into redox homeostasis regulation limits the appropriate clinical application in multiple aspects with medication dose range as the main concern.
5. The molecular signaling mechanisms of NAC in seeding cells and biomaterials is of great importance especially for mammalian models due to the envisaged application of the studied biomaterials concerning the complex *in vivo* environment. However, the scarcity of relevant literature and review reports makes it difficult to guide the clinical practice of biomaterials.
