# Supplementary material for: The role of N-acetylcysteine in osteogenic microenvironment for bone tissue engineering
Source: Front Cell Dev Biol. 2024 Jul 11;12:1435125. doi: 10.3389/fcell.2024.1435125 (PMC11269162; doi:10.3389/fcell.2024.1435125)
Supplement: Supplementary file 1 [file DataSheet1.docx]

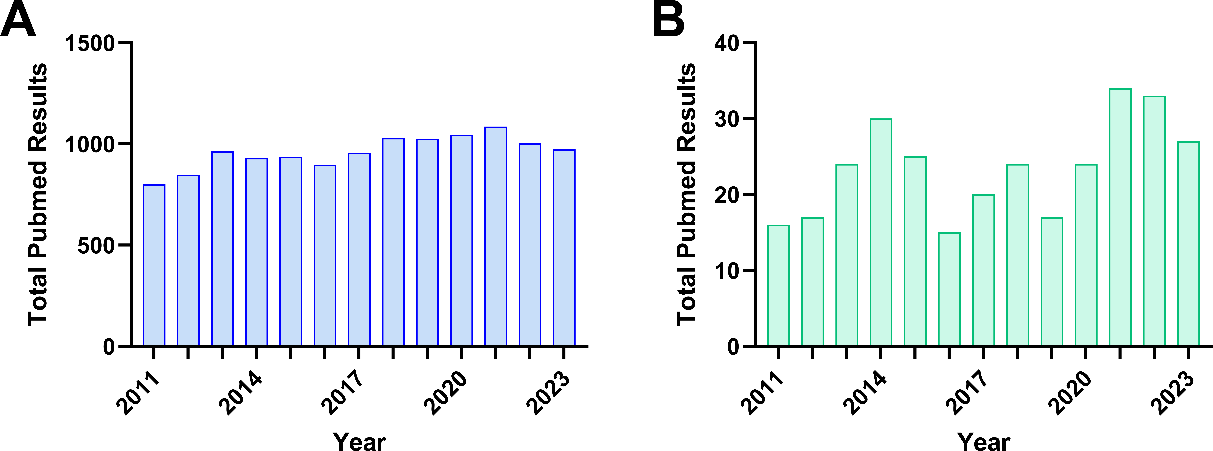


Figure S1. Number of NAC-related publications from 2011 to 2023. (A) Yearly total number of PubMed-listed publications with “N-acetylcysteine” or “N-acetyl-L-cysteine” or “acetylcysteine” in the title and/or abstract. (B) Yearly total number of PubMed-listed publications with “N-acetylcysteine” or “N-acetyl-L-cysteine” or “acetylcysteine” and “bone” in the title and/or abstract.
